# Impact of Neoadjuvant Immunotherapy on Recurrence-Free Survival in Patients with High-Risk Localized HCC

**DOI:** 10.1158/2767-9764.CRC-24-0151

**Published:** 2024-08-15

**Authors:** Mari Nakazawa, Mike Fang, Tyrus Vong, Jane Zorzi, Paige Griffith, Robert A. Anders, Kiyoko Oshima, Amy K. Kim, Jacqueline Laurin, Kelly J. Lafaro, Christopher R. Shubert, William R. Burns, Jin He, Richard A. Burkhart, Benjamin Philosophe, Jeffrey Meyer, Robert P. Liddell, Christos Georgiades, Kelvin Hong, Won Jin Ho, Marina Baretti, Alexandra T. Strauss, Mark Yarchoan

**Affiliations:** 1 Sidney Kimmel Comprehensive Cancer Center, Johns Hopkins University School of Medicine, Baltimore, Maryland.; 2 Department of Gastroenterology and Hepatology, Johns Hopkins University School of Medicine, Baltimore, Maryland.; 3 Department of Pathology, Johns Hopkins University School of Medicine, Baltimore, Maryland.; 4 Department of Surgery, Johns Hopkins University School of Medicine, Baltimore, Maryland.; 5 Department of Radiation Oncology and Molecular Radiation Sciences, Johns Hopkins University School of Medicine, Baltimore, Maryland.; 6 Department of Interventional Radiology, Johns Hopkins University School of Medicine, Baltimore, Maryland.

## Abstract

Surgical resection for localized hepatocellular carcinoma (HCC) is typically reserved for a minority of patients with favorable tumor features and anatomy. Neoadjuvant immunotherapy can expand the number of patients who are candidates for surgical resection and potentially reduce the chance for recurrence, but its role in HCC not defined. We retrospectively examined the outcomes of patients who underwent surgical resection for HCC at the Johns Hopkins Hospital and compared the clinical outcomes of patients who received neoadjuvant immunotherapy with those who underwent upfront resection. The clinical cohort included a total of 92 patients, 36 of whom received neoadjuvant immune checkpoint inhibitor (ICI)-based treatment. A majority of patients (61.1%) who received neoadjuvant ICI–based therapy were outside of standard resectability criteria and were more likely to have features known to confer risk of disease recurrence, including α-fetoprotein ≥ 400 ng/mL (*P* = 0.02), tumor diameter ≥ 5 cm (*P* = 0.001), portal vein invasion (*P* < 0.001), and multifocality (*P* < 0.001). Patients who received neoadjuvant immunotherapy had similar rates of margin-negative resection (*P* = 0.47) and recurrence-free survival (RFS) as those who underwent upfront surgical resection (median RFS 44.8 months compared with 49.3 months, respectively, log-rank *P* = 0.66). There was a nonsignificant trend toward superior RFS in the subset of patients with a pathologic response (tumor necrosis ≥ 70%) with neoadjuvant immunotherapy. Neoadjuvant ICI-based therapy may allow high-risk patients, including those who are outside traditional resectability criteria, to achieve comparable clinical outcomes with those who undergo upfront resection.

**Significance:**

Surgical resection for localized HCC is typically only reserved for those with solitary tumors without vascular invasion. In this retrospective analysis, we show that neoadjuvant immunotherapy may allow high-risk patients, including those who are outside of standard resection criteria, to undergo successful margin-negative resection and achieve comparable long-term clinical outcomes compared with upfront resection. These findings highlight need for prospective studies on neoadjuvant immunotherapy in HCC.

## Introduction

Hepatocellular carcinoma (HCC) accounts for 80% of all primary liver cancers and is currently the fifth leading cause of cancer mortality in the United States (1), with mortality closely matching incidence (2). Although the burden of HCC is variable by country, recent projections suggest that the worldwide incidence of liver cancer will continue to rise (3, 4), with the epidemiology of underlying liver disease shifting away from viral hepatitis and toward metabolic dysfunction-associated steatohepatitis (5). Only approximately 30% of patients diagnosed with HCC are considered eligible for resection by current Western guidelines due to the presence of extrahepatic and vascular extensions, poor hepatic reserve, and/or anatomical considerations (including multinodular disease) that preclude adequate resection margins (6–8). Even in those who undergo curative-intent resection, long-term outcomes remain poor, with a vast majority of patients recurring within 5 years of surgery (9–11), stemming from the presence of micrometastatic disease at the time of surgery and/or new foci of disease developing in the cirrhotic liver. Perioperative systemic therapy for HCC is an area of active clinical investigation with the potential to reduce micrometastatic disease burden and in turn improve postresection outcomes.

Multiple novel immunotherapy-based regimens have demonstrated improved survival versus sorafenib in unresectable HCC and expanded the vista of therapeutic options (12, 13). The impact of immune checkpoint inhibitor (ICI) therapy on the treatment landscape of advanced HCC has led to an intense interest in investigating its potential role in earlier stages of HCC. Recently, immunotherapy-based combinations have been shown to improve recurrence-free survival (RFS) in high-risk patients following curative-intent resection or ablation, although longer follow-up is needed to assess its impact on overall survival (OS; refs. 14, 15). In addition, multiple single-institution clinical trials have explored the feasibility of neoadjuvant immunotherapy (16, 17). Collectively, available studies of neoadjuvant immunotherapy in HCC show that this approach is feasible, including in those with high-risk tumors who were poor candidates for upfront resection, with pathologic response rates of 20% to 33% (18–20). Whether neoadjuvant immunotherapy can improve long-term survival outcomes is unknown. We hypothesize that patients who undergo neoadjuvant ICI treatment have higher-risk tumor features compared with those who undergo upfront surgery but that neoadjuvant ICI may allow for such high-risk patients to achieve comparable survival outcomes.

## Methods

### Patient cohort

We retrospectively identified 95 patients who underwent liver resection at the Johns Hopkins Hospital between January 1, 2017, and December 1, 2023, with a pathologically confirmed diagnosis of HCC. Electronic medical records were reviewed to determine demographic characteristics, clinical features, laboratory values prior to initiation of neoadjuvant ICI–based therapy or surgery (whichever occurred first), treatment history (in particular, receipt of neoadjuvant ICI therapy), tumor characteristics based on pathology of resected tumor specimen, and clinical outcomes. The primary clinical endpoints of interest included RFS (defined as time from curative-intent hepatectomy to radiographic disease recurrence or death due to any cause) and OS (defined as time from curative-intent hepatectomy to death due to any cause). Data cut-off was set as January 3, 2024. This study was approved by the Johns Hopkins Institutional Review Board (#00417376) and performed in accordance with the U.S. Common Rule.

Our institutional multidisciplinary treatment practice patterns evaluate patients for resection who are outside of traditional resection criteria established by the Barcelona Clinic Liver Cancer (BCLC) Staging System (21), but usually only after a period of systemic therapy. Since the advent of effective combination systemic therapies, our institution has offered resection to patients with high-risk tumor features following neoadjuvant therapy who previously were not considered for upfront resection. Since 2017, our institution has prospectively enrolled patients on neoadjuvant clinical trials of anti-PD1–based systemic therapies [including cabozantinib plus nivolumab (NCT03299946) and nivolumab alone or in combination with lymphocyte activation gene-3 (LAG3) inhibitor relatlimab (NCT04658147)]. These clinical trials specifically enabled patients outside of traditional Western resection criteria to enroll, including patients with multifocal disease, locally advanced disease, and portal vein invasion. Whereas resectability in localized HCC is defined not only by the likelihood of achieving a margin-negative (R0) resection but also by the safety of the surgery in the cirrhotic liver and the extent of high-risk disease features (e.g., vascular invasion), upfront resectability is defined in this analysis according to the BCLC criteria of BCLC A disease with a solitary lesion (22).

Patients treated according to our various study protocols, as well as patients treated with systemic therapy off-protocol, were both included in the primary analysis. Mixed phenotypic tumors (e.g., mixed HCC/cholangiocarcinoma) were excluded from the present analysis. Patients who did not undergo curative-intent hepatectomy or had metastatic disease identified during surgery that was previously unrecognized, were excluded. Patients who had combination locoregional therapy and local therapies (e.g., transarterial chemoembolization/transarterial radioembolization; Y90) were included in the overall cohort but not included in the analysis for the effect of pathologic response on RFS in the neoadjuvant immunotherapy treatment arm if they received the local therapy following immunotherapy because of an inability to attribute pathologic responses to immunotherapy alone. Inclusion and exclusion criteria are further outlined in Supplementary Fig. S1.

### Statistical methods

We applied univariate Cox proportional-hazards survival models to assess effect sizes and generate HR based on demographic and tumor-specific features. Kaplan–Meier survival curves were constructed to compare survival between various clinical cohorts, including neoadjuvant ICI–treated and untreated patients, with significance determined by the two-sided log-rank test. Differences between categorical variables were assessed using the Fisher exact test, whereas those between numeric variables were assessed using the Mann–Whitney U test.

### Data availability

The data generated in this study are available upon request from the corresponding author.

## Results

The final clinical cohort included a total of 92 patients, 36 of whom underwent neoadjuvant ICI–based treatment (Table 1). The overall cohort was predominantly male (69.6%), White (57.6%), with preserved liver function (98.9% Child Pugh A). Of those who received neoadjuvant ICI (Table 2), a majority were treated with anti-PD1–based therapy, either as monotherapy (27.8%), in combination with tyrosine kinase inhibitor (36.1%), or in combination with anti-LAG3 (16.7%). A majority of those treated with neoadjuvant ICI were treated under a clinical trial protocol (69.4%) for at least two cycles. Those who received neoadjuvant ICI–based treatment were more likely to have higher-risk disease, as indicated by the greater proportion of patients who had α-fetoprotein (AFP) ≥ 400 ng/mL at baseline (38.9% vs. 14.3%, Fisher exact test *P* = 0.02), large tumors greater than 5 cm (72.2% vs. 37.5%, Fisher exact test *P* = 0.001), portal vein invasion (25.0% vs. 0%, Fisher exact test *P* < 0.001), and multiple tumor foci (50.0% vs. 12.5% multiple tumors, Fisher exact test *P* < 0.001). Most patients (83.3%) treated with neoadjuvant ICI had stable disease by RECIST version 1.1 as the best response to therapy prior to surgery, with a minority (13.9%) achieving partial response. In both cohorts, a comparable minority of patients received local therapy prior to surgery. Twelve (33.3%) patients in the neoadjuvant ICI–treated cohort received adjuvant therapy, most commonly in the form of anti-PDL1 monotherapy.

**Table 1:** Baseline characteristics of patients who underwent ICI-based neoadjuvant therapy or upfront resection for HCC

	Neoadjuvant ICI	Upfront surgery	*P* value
	(*N* = 36)	(*N* = 56)	
Age at surgery (years)			
Mean (SD)	63.3 (12.1)	67.3 (10.6)	0.179
Median [Min, Max]	65.0 [23.0, 78.0]	68.5 [30.0, 87.0]	
Gender			
Male	21 (58.3%)	43 (76.8%)	0.0683
Female	15 (41.7%)	13 (23.2%)	
Race			
White	25 (69.4%)	28 (50.0%)	0.156
Black	7 (19.4%)	18 (32.1%)	
Asian	4 (11.1%)	6 (10.7%)	
Other	0 (0%)	4 (7.1%)	
Etiology of liver disease			
Viral	16 (44.4%)	22 (39.3%)	0.858
ETOH	1 (2.8%)	4 (7.1%)	
Viral/ETOH	1 (2.8%)	3 (5.4%)	
MASH/MASLD	8 (22.2%)	11 (19.6%)	
Other	5 (13.9%)	5 (8.9%)	
None	5 (13.9%)	11 (19.6%)	
Child–Pugh			
A	36 (100%)	55 (98.2%)	1
B	0 (0%)	1 (1.8%)	
ALBI grade			
1	30 (83.3%)	46 (82.1%)	1
2/3	6 (16.7%)	10 (17.9%)	
AFP ≥ 400 ng/mL			
No	22 (61.1%)	43 (76.8%)	0.0231
Yes	14 (38.9%)	8 (14.3%)	
Missing	0 (0%)	5 (8.9%)	
Largest tumor ≥ 5 cm			
No	10 (27.8%)	35 (62.5%)	0.0014
Yes	26 (72.2%)	21 (37.5%)	
Portal vein invasion			
No	27 (75.0%)	56 (100%)	<0.001
Yes	9 (25.0%)	0 (0%)	
Multifocal disease			
No	18 (50.0%)	49 (87.5%)	<0.001
Yes	18 (50.0%)	7 (12.5%)	
Received neoadjuvant local tx			
No	24 (66.7%)	43 (76.8%)	0.34
Yes	12 (33.3%)	13 (23.2%)	
Received adjuvant tx			
No	24 (66.7%)	54 (96.4%)	<0.001
Yes	12 (33.3%)	2 (3.6%)	

Differences between categorical variables were assessed using the Fisher exact test, whereas those between numeric variables were assessed using the Mann–Whitney U test.

Abbreviations: ALBI, albumin–bilirubin; ETOH, alcohol; ICI, immune checkpoint inhibitor; MASH, metabolic dysfunction–associated steatohepatitis; MASLD, metabolic dysfunction–associated steatotic liver disease; tx, treatment.

**Table 2:** Detailed baseline characteristics in the ICI-treated cohort by whether tumor was resectable at baseline according to BCLC criteria

	Resectable	Unresectable	*P* value
	(*N* = 14)	(*N* = 22)	
Age at surgery (years)			
Mean (SD)	63.6 (9.98)	63.1 (13.6)	0.884
Median [Min, Max]	64.0 [44.0, 77.0]	68.0 [23.0, 78.0]	
Gender			
Male	9 (64.3%)	12 (54.5%)	0.732
Female	5 (35.7%)	10 (45.5%)	
Etiology of liver disease			
Viral	9 (64.3%)	7 (31.8%)	0.272
ETOH	0 (0%)	1 (4.5%)	
Viral/ETOH	0 (0%)	1 (4.5%)	
MASH/MASLD	3 (21.4%)	5 (22.7%)	
Other	0 (0%)	5 (22.7%)	
None	2 (14.3%)	3 (13.6%)	
ALBI grade			
1	13 (92.9%)	17 (77.3%)	0.37
2/3	1 (7.1%)	5 (22.7%)	
BCLC staging			
A	14 (100%)	1 (4.5%)	<0.001
B	0 (0%)	12 (54.5%)	
C	0 (0%)	9 (40.9%)	
ICI regimen			
Anti-PD1	4 (28.6%)	6 (27.3%)	0.78
Anti-PD1/TKI	7 (50.0%)	6 (27.3%)	
Anti-PD1/anti-LAG3	2 (14.3%)	4 (18.2%)	
Anti-PDL1/anti-VEGF	1 (7.1%)	3 (13.6%)	
Anti-PD(L)1/anti-CTLA4	0 (0%)	2 (9.1%)	
Anti-CTLA4/TKI	0 (0%)	1 (4.5%)	
Duration of ICI (days)			
Mean (SD)	48.4 (32.6)	62.0 (59.9)	0.495
Median [Min, Max]	37.0 [0, 112]	42.0 [0, 258]	
AFP ≥ 400 ng/mL			
No	9 (64.3%)	13 (59.1%)	1
Yes	5 (35.7%)	9 (40.9%)	
Best response by RECIST			
PD	1 (7.1%)	0 (0%)	0.327
SD	12 (85.7%)	18 (81.8%)	
PR	1 (7.1%)	4 (18.2%)	
Largest tumor ≥ 5 cm			
No	4 (28.6%)	6 (27.3%)	1
Yes	10 (71.4%)	16 (72.7%)	
Portal vein invasion			
No	14 (100%)	13 (59.1%)	0.00601
Yes	0 (0%)	9 (40.9%)	
Multifocal disease			
No	14 (100%)	4 (18.2%)	<0.001
Yes	0 (0%)	18 (81.8%)	
Treated on clinical trial			
No	3 (21.4%)	8 (36.4%)	0.467
Yes	11 (78.6%)	14 (63.6%)	
Major path response			
No	12 (85.7%)	12 (54.5%)	0.0756
Yes	2 (14.3%)	10 (45.5%)	

Differences between categorical variables were assessed using the Fisher exact test, whereas those between numeric variables were assessed using the Mann–Whitney U test.

Duration of ICI indicates time (in days) from the first to the final dose of neoadjuvant ICI therapy.

Abbreviations: ALBI, albumin–bilirubin; BCLC, Barcelona Clinic Liver Cancer; CTLA4, cytotoxic T-lymphocyte–associated protein 4; ETOH, alcohol; MASH, metabolic dysfunction–associated steatohepatitis; MASLD, metabolic dysfunction–associated steatotic liver disease; PD, progressive disease; PD1, programmed cell death protein 1; PDL1, programmed cell death ligand 1; PR, partial response; RECIST, Response Evaluation Criteria in Solid Tumors; SD, stable disease; TKI, tyrosine kinase inhibitor; VEGF, vascular endothelial growth factor.

**Figure 1:**
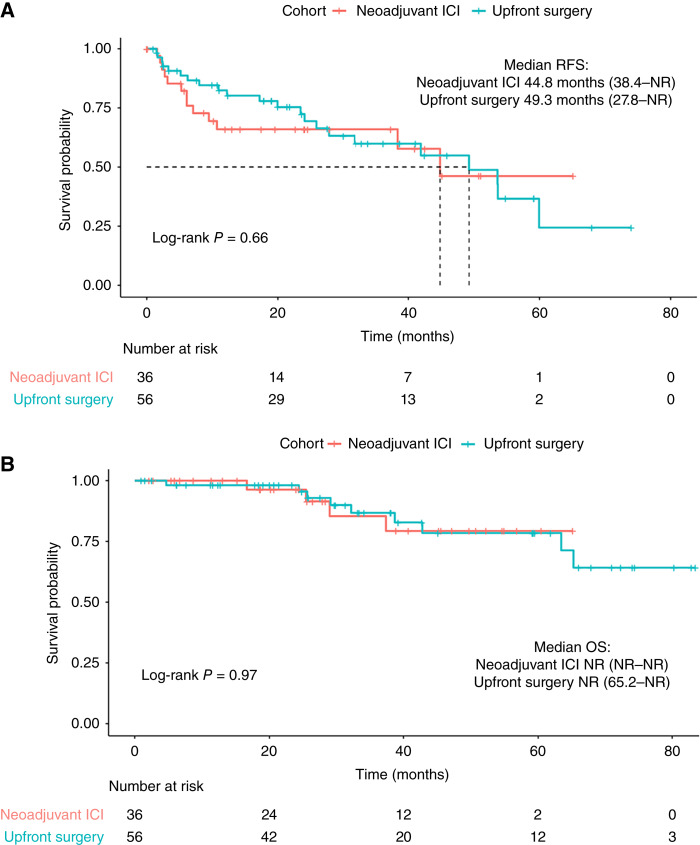
**A,** Kaplan–Meier survival curve RFS of patients with resectable HCC who underwent neoadjuvant ICI therapy and upfront surgery. **B,** Kaplan–Meier survival curve depicting OS of same cohorts.

**Figure 2:**
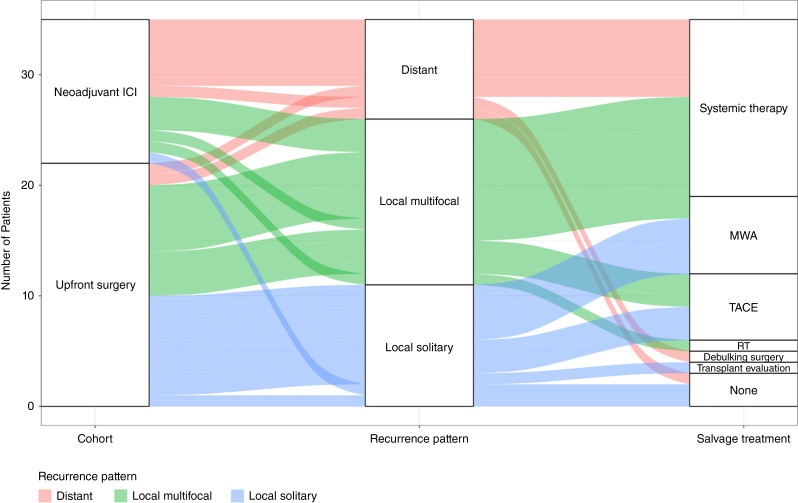
Alluvial diagram depicting patterns of recurrence and subsequent line of therapy in patients with recurrence in patients treated with upfront surgery and neoadjuvant ICI. MWA, microwave ablation; TACE, transarterial chemoembolization, RT, radiation therapy.

We first sought to determine the impact of neoadjuvant ICI on long-term outcomes. The median RFS in the ICI-treated cohort was 44.8 months [95% confidence interval (CI), 38.4–not reached, (NR)] compared with 49.3 months in the upfront surgery cohort (95% CI, 27.8–NR); log-rank *P* = 0.66 (Fig. 1A). The median OS has not been reached in both cohorts; log-rank *P* = 0.97 (Fig. 1B). We also evaluated the patterns of recurrence for patients who recurred in both cohorts (Fig. 2) and observe that neoadjuvant ICI–treated patients more commonly had distant recurrences.

**Figure 3:**
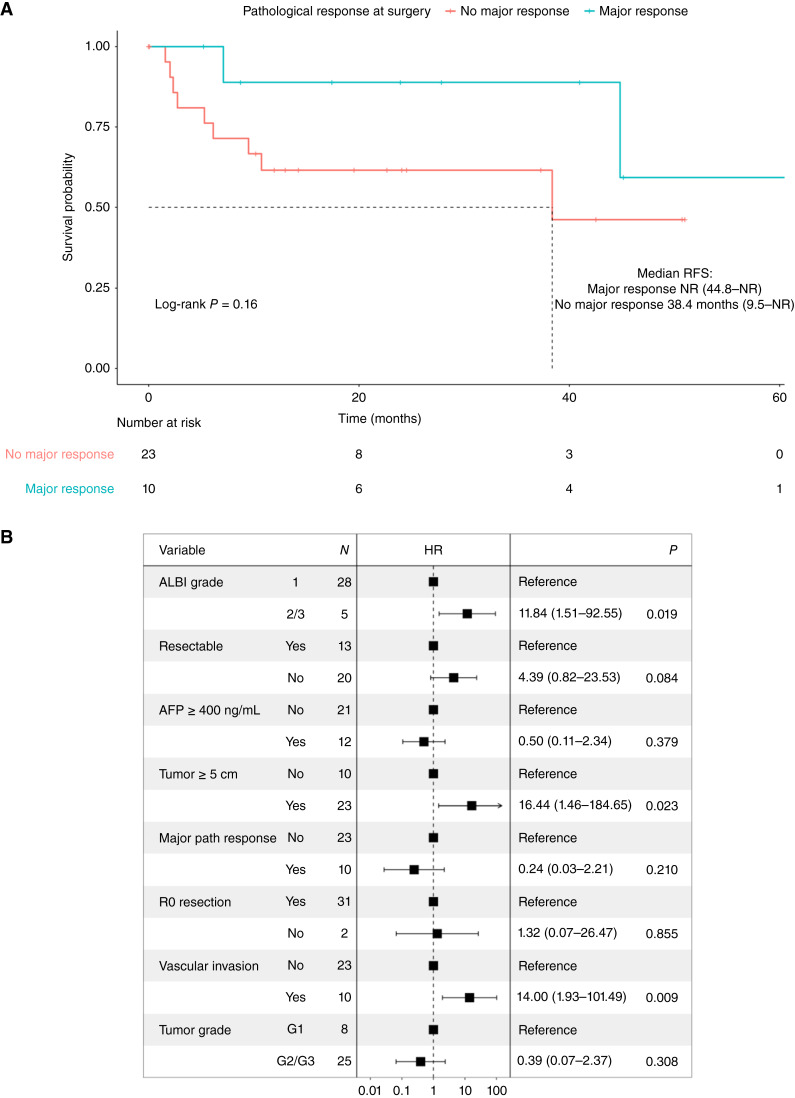
**A,** RFS in the neoadjuvant ICI–treated cohort by the presence of major pathologic response at time of surgery, defined as tumor necrosis ≥70%. Three patients who underwent neoadjuvant locoregional procedures following the receipt of ICI were omitted. **B,** Forest plot depicting HR for RFS following resection in the neoadjuvant ICI–treated cohort. Resectability is as defined by current BCLC staging. Vascular invasion indicates the presence of micro/macrovascular invasion on final pathology. ALBI, albumin–bilirubin; major path response, major pathologic response at the time of surgery, defined as tumor necrosis ≥70%.

In patients treated with neoadjuvant ICI who did not undergo liver-directed local therapy following ICI treatment, 10/33 (30.3%) patients had a major pathologic response at the time of resection, defined as tumor necrosis ≥70%. In contrast, 6/33 (18.2%) had a minor pathologic response (necrosis 30%–69%) and 17/33 (51.5%) had no pathologic response (necrosis 0%–29%). Patients who achieved a major pathologic response at the time of surgery had numerically superior RFS compared with those with a minor response (median NR vs. 38.3 months, log-rank *P* = 0.16, Fig. 3A). In a multivariable univariate Cox regression, a major pathologic response was not predictive of RFS (HR 0.62, 95% CI, 0.03–11.03) when controlled for variables, including albumin–bilirubin (ALBI) grade, AFP ≥ 400 ng/mL, tumor size ≥ 5 cm, tumor focality, R0 resection status, vascular invasion on final pathology, and tumor grade. Upfront resectability status was negatively prognostic for RFS (HR 4.39, 95% CI, 0.82–23.53) but did not reach statistical significance. ALBI grades 2 and 3, tumor size ≥ 5 cm, and the presence of vascular invasion on final pathology were negative predictors for RFS in the ICI-treated cohort (Fig. 3B).

**Figure 4:**
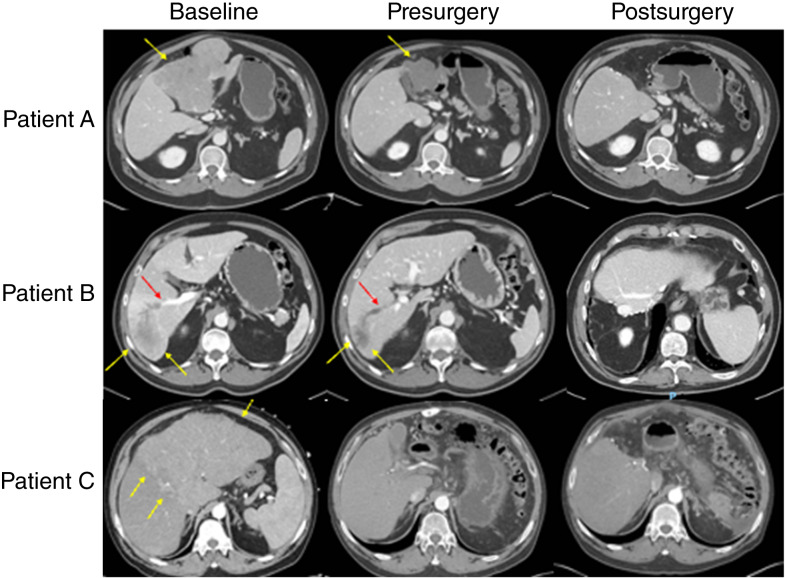
Baseline, presurgery, and postsurgery contrast-enhanced CT images of three BCLC C patients (vascular invasion present) treated with neoadjuvant ICI–based therapy with successful R0 resection. Patient A: CT venous phase imaging showing a large left-liver mass measuring 13.1 cm (yellow arrow) with associated occlusion of the left portal vein (not shown) at baseline. Patient B: CT venous phase imaging showing a 5.3-cm mass with necrosis (yellow arrows) and tumor thrombus within the right portal vein (red arrow); additional multifocal disease (not shown) at baseline. Patient C: CT arterial phase imaging showing large infiltrative lesion involving entirety of the left liver at baseline (yellow arrows) and associated left portal vein invasion (not shown), with complete radiographic response to ICI therapy prior to surgery.

In the total cohort of neoadjuvant ICI–treated patients, 22/36 (61.1%) patients were outside of traditional resectability criteria due to multifocal tumors or vascular invasion. In particular, nine (25.0%) patients had BCLC C disease with evidence of portal vein invasion. Nevertheless, a vast majority of patients (94.4%) were able to undergo successful R0 resection, comparable with that of patients who underwent upfront surgery. Detailed pathologic outcomes in both subgroups are outlined in Table 3. Figure 4 illustrates CT images three of such patients at baseline, following completion of neoadjuvant ICI therapy, and following successful resection.

**Table 3:** Pathologic outcomes at time of surgery in patients who underwent ICI-based neoadjuvant therapy or upfront resection for HCC

	Neoadjuvant ICI	Upfront surgery	*P* value
	(*N* = 36)	(*N* = 56)	
Size of largest viable tumor (cm)			
Mean (SD)	6.06 (4.74)	5.92 (4.51)	0.917
Median [Min, Max]	5.20 [0, 15.0]	4.45 [0, 18.0]	
R0 resection			
Yes	34 (94.4%)	49 (87.5%)	0.474
No	2 (5.6%)	7 (12.5%)	
Closest margin (mm)			
Mean (SD)	9.16 (13.0)	7.55 (9.03)	0.527
Median [Min, Max]	5.00 [0, 72.0]	6.00 [0, 47.0]	
Missing	3 (8.3%)	3 (5.4%)	
Vascular invasion			
No	25 (69.4%)	36 (64.3%)	0.657
Yes	11 (30.6%)	20 (35.7%)	
Grade			
G1	8 (22.2%)	16 (28.6%)	0.685
G2	20 (55.6%)	33 (58.9%)	
G3	5 (13.9%)	5 (8.9%)	
Missing	3 (8.3%)	2 (3.6%)	
T staging			
T1	14 (38.9%)	31 (55.4%)	0.132
T2	13 (36.1%)	18 (32.1%)	
T3	5 (13.9%)	4 (7.1%)	
TX	4 (11.1%)	1 (1.8%)	
T4	0 (0%)	2 (3.6%)	

Differences between categorical variables were assessed using the Fisher exact test, whereas those between numeric variables were assessed using the Mann–Whitney U test.

## Discussion

We report long-term clinical outcomes of patients treated with neoadjuvant ICI–based therapy and a contemporaneous cohort of those who underwent upfront resection for HCC at a single academic institution. The patients who received neoadjuvant ICI therapy were generally patients who had tumors that were deemed high-risk for upfront resection due to large tumor size, proximity to critical structures, multifocality, or suspected macrovascular invasion. Conversely, the majority of patients in the upfront surgery cohort had solitary lesions and would generally have received upfront resection under current BCLC guidelines. We observe that that whereas most patients who underwent neoadjuvant therapy fell outside of traditional BCLC criteria for resection, the RFS in those who received neoadjuvant ICI therapy was comparable with those who underwent upfront surgery.

These data reflect the clinical practices of the Johns Hopkins Liver Multidisciplinary Clinic, where patients with HCC outside of conventional resection criteria are often considered for upfront systemic therapy or locoregional therapies (including transarterial chemoembolization/transarterial radioembolization, Y90, and radiation therapy). Although many factors are considered, in general, patients who are deemed to be unresectable upfront with macrovascular invasion and/or larger tumor burdens, as reflected by extremely high AFPs (i.e., >10,000 ng/mL) or large tumors (i.e., >10 cm), are more likely to be offered systemic therapy. Notably, although prior studies have reflected on the feasibility of resection on patients with features such as large tumor size, multinodular disease, and major vascular invasion, outcomes were inferior compared with those falling under traditional resectability criteria (23). Our observation that RFS was comparable in the ICI-treated and untreated cohorts provides initial evidence that neoadjuvant ICI–based therapy may be effective in transforming the natural histories of these patients postresection to that of one comparable with those who received upfront surgery for lower-risk HCC. However, larger prospective studies are needed to confirm that neoadjuvant immunotherapeutic strategies can expand the pool of patients who should be considered for resection.

Several neoadjuvant and perioperative ICI-based studies have demonstrated that single and combination ICI therapy is well tolerated, with a vast majority of patients undergoing successful resection and some observations suggesting that the presence of a major pathologic response at the time of surgery may predict recurrence (18–20). However, given that the study of neoadjuvant ICI–based therapy in HCC is still in its relative infancy, the optimal endpoint for neoadjuvant investigations that translates to a change in the natural disease trajectory of resected HCC remains unclear. Our results suggest that upfront resectability status was a negative prognostic factor for RFS, although not statistically significant. Furthermore, patients with a major pathologic response (i.e., ≥70% necrosis) at the time of surgery had numerically superior RFS. Although there seems to be a correlation, the threshold of tumor necrosis as it translates to improved survival outcomes remains under active investigation. In addition, the role of adjuvant ICI remains relevant given the observation that more distant recurrences occurred in the neoadjuvant ICI–treated cohort, underscoring the need for systemic control.

Due to the retrospective nature of this analysis, there are several limitations to consider with the interpretation of these results. We cannot exclude the possibility that differences between the lower-risk upfront surgery and higher-risk neoadjuvant cohorts are the reason why no difference was observed in their clinical outcomes. For example, we note that although the collected demographic characteristics of the cohorts were largely comparable, a numerically higher proportion of Black patients were in the upfront surgery cohort. This difference could be related to patient-level factors or comorbidities affecting neoadjuvant clinical trial eligibility, which in turn affected HCC outcomes. The true effect size for the impact of neoadjuvant ICI therapy is difficult to estimate without a similarly high-risk group of patients who underwent upfront surgery, which is typically not undertaken in the standard-of-care management of HCC. In addition, patients who underwent neoadjuvant ICI–based therapy as described in this article does not represent the experience of all patients who were treated with neoadjuvant ICI regimens because only those who then underwent resection were included. A small minority of patients treated in the neoadjuvant trials represented in this analysis were ultimately unable to undergo surgery, most commonly due to the presence of more extensive disease than what was recognized. Finally, our single-institution experience may not be applicable to other centers due to differences in institutional practice and patient populations. Despite these limitations, this is the largest retrospective cohort to report on the outcomes of neoadjuvant ICI–treated and untreated patients in a rapidly evolving landscape of ICI-based treatments available in HCC.

### Conclusions

Although patients treated with neoadjuvant ICI–based therapy for HCC in this cohort had higher-risk disease features and generally would not have been considered surgical candidates under BCLC criteria, they achieved long-term outcomes comparable with a cohort of patients who underwent upfront surgery. Our observations highlight the need for future prospective trials to further defined the role of neoadjuvant ICI therapy in both traditionally resectable and high-risk localized HCC populations.

## Supplementary Material

Supplementary Figure 1 S1. Flow diagram depicting inclusion and exclusion criteria for current study
