# Supplementary material for: Impact of Neoadjuvant Immunotherapy on Recurrence-Free Survival in Patients with High-Risk Localized HCC
Source: Cancer Res Commun. 2024 Aug 15;4(8):2123–32. doi: 10.1158/2767-9764.CRC-24-0151 (PMC11324369; doi:10.1158/2767-9764.CRC-24-0151)
Supplement: Supplementary Figure 1 — S1. Flow diagram depicting inclusion and exclusion criteria for current study [file crc-24-0151_supplementary_figure_1_suppsf1.pdf]

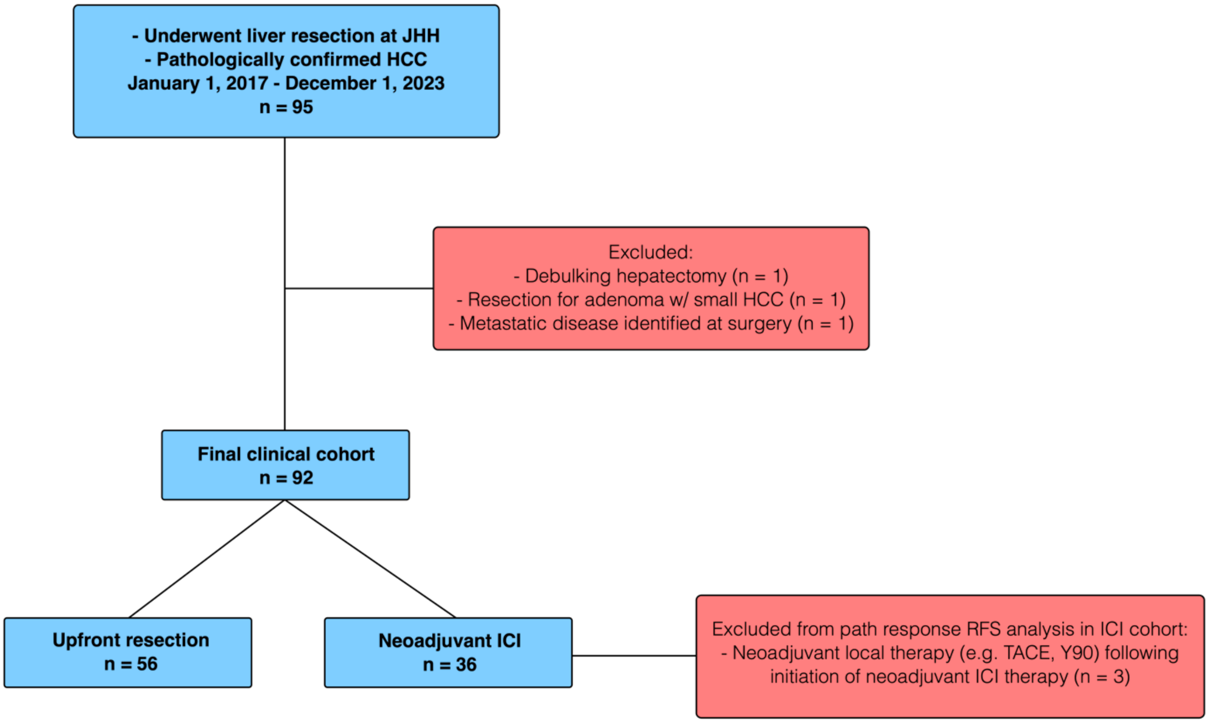

Supplemental Figure 1: Flow diagram depicting inclusion and exclusion criteria for current study. HCC, hepatocellular carcinoma; JHH, Johns Hopkins Hospital; ICI, immune checkpoint inhibitor; TACE, transarterial chemoembolization; RFS, recurrence-free survival.
